# The Response of Monoecious and Dioecious Cultivars of Agricultural Hemp to an Organic Fertiliser Derived from Black Soldier Fly Frass

**DOI:** 10.3390/insects16090918

**Published:** 2025-09-02

**Authors:** Gordon Kavanagh, Susanne Schilling, Rainer Melzer, Simon Hodge

**Affiliations:** 1 School of Biology and Environmental Science, University College Dublin, D04 Dublin, Ireland; susanne.schilling@ucd.ie (S.S.); rainer.melzer@ucd.ie (R.M.); 2 School of Agriculture and Food Science, University College Dublin, D04 Dublin, Ireland; simon.hodge@ucd.ie

**Keywords:** *Cannabis sativa*, *Hermetia illucens*, insect farming, circular economy

## Abstract

The development of sustainable food systems requires the appraisal of novel methods of maintaining soil nutrients and the evaluation of alternative crops and sward systems. Accordingly, this study examined how agricultural hemp responded to an organic fertiliser produced from black soldier fly (BSF; *Hermetia illucens*) frass. The results indicate that at equivalent N-rates (approx. 0.16 g N per pot), a commercial BSF frass fertiliser (“HexaFrass”) produced similar increases in shoot growth of monoecious and dioecious hemp seedlings as was achieved with established organic fertilisers (chicken manure; Miracle-gro). In one cultivar, CFX-2, it was shown that positive effects on shoot growth could be achieved at low fertiliser rates (≤4 g per pot), and additional fertiliser did not induce additional shoot growth. Application of HexaFrass increased the shoot content of P, K, and Mg but not N, an important consideration if hemp straw and foliage are to be used as livestock feed. Insect frass fertilisers can supply hemp seedlings with the nutrients required for accelerated shoot growth, but further work is needed to evaluate different fertiliser regimes (e.g., split application; liquid extracts) and to assess how frass fertilisers affect yield and chemical profiles of hemp seeds.

## Simple Summary

To make food production more sustainable, farmers are investigating novel ways to maintain soil nutrient levels and are exploring new or alternative crops. This investigation examined how one possible alternative crop, agricultural hemp, responded to a novel organic fertiliser produced from insect frass, the waste produced from insect farming. Insect frass contains all major plant nutrients (N:P:K) in addition to bioactive compounds, such as chitin, which occurs in insect exoskeletons. Our results indicated that “HexaFrass”, a frass fertiliser produced from black soldier flies, increased shoot growth in six different cultivars and in both male and female hemp plants and had similar effects on hemp growth to those obtained with other standard organic fertilisers. The increases in shoot growth occurred at relatively low HexaFrass application rates, and additional fertiliser did not result in additional shoot growth. Insect frass fertilisers are based on the repurposing of biological wastes into new commodities and therefore have potential to enhance the sustainability of farming within circular economy frameworks.

## 1. Introduction

To maximise yields, many Irish arable, livestock, and horticultural systems remain heavily reliant on petrochemical-based fertilisers and synthetic pesticides. These highly productive farming systems are, however, of growing concern because of the ecological damage and environmental harm they cause [1,2]. Consequently, Irish farmers and researchers are exploring several areas where food and fibre production could be made more sustainable and the overall environmental footprint of Irish farms could be reduced [3,4]. From an agricultural perspective, avenues being investigated include the consideration of livestock genetics, reduction of greenhouse gas emissions, inclusion of nitrogen-fixing legumes into grazing systems, growing of new crop varieties and/or archaic cereals, and exploration of alternative amendments to maintain soil nutrient levels [5].

Organically derived fertilisers can help meet plant nutrient demands whilst also enhancing soil carbon and improving soil microbiota and invertebrate assemblages [6,7]. Traditional organic fertilisers encompass manures (mammal and avian), composts, green manures, and cover crops, with other types of animal-derived ‘meals’ (e.g., fish, bone, poultry) also considered as organic alternatives. In addition to these long-established amendments, novel organic inputs have grown in popularity in recent years, including compost “teas”, humic acids, and seaweed-derived biostimulants [8]. Fertilisers based on insect frass are a by-product of insect farming, with the ‘frass’ typically consisting of insect excreta, shed exoskeletons, uneaten diet, various body parts, microorganisms, and bioactive compounds such as chitin [9,10,11,12]. As these frass fertilisers involve the repurposing of biological wastes into new commodities (insect protein feed and fertiliser products), they are frequently seen as exemplars for the advancement of circular economy models connecting waste management and sustainable food production [11,12,13].

Insect frass fertilisers (IFFs) are now produced as a by-product from the farming of several insect species, including yellow mealworms (*Tenebrio molitor* L.), lesser mealworms (*Alphitobius diaperinus* Panzer), house crickets (*Acheta domesticus* L.), and tropical crickets (*Gryllodes sigillatus* F. Walker). In vegetables, herbs and fodder plants, frass fertiliser produced by the black soldier fly (*Hermetia illucens* L.) has been shown to produce equivalent plant growth and yields as synthetic fertilisers and traditional organic fertilisers such as chicken manure pellets [14,15,16,17]. Black soldier fly fertiliser frass fertiliser (BSFF) has also been shown to increase the growth and yield of pasture grasses such as perennial ryegrass (*Lolium perenne* L.), Timothy (*Phleum pratense* L.), and cocksfoot (*Dactylis glomerata* L.), and cereals such as maize (*Zea mays* L.), barley (*Hordeum vulgare* L.), and oats (*Avena sativa* L.) [18,19,20,21,22]. These positive effects tend to be more apparent when the BSFF is applied to nutrient-poor soils and at low to medium application rates, with high application rates sometimes resulting in reduced shoot growth or even seedling mortality [21]. Studies have also reported variation in the occurrence, or magnitude, of plant responses to insect frass fertilisers, even when trials appear to be carried out under the same conditions [16,22].

The primary mechanism behind the positive effects of IFFs on plant growth is provision of macro- and micro-nutrients [9]. Chemical analysis of several BSFFs indicated average N-P-K values of around 3.2-1.2-2.9, with other macronutrients (Mg, Na, Ca, S) occurring at approximately 0.5% by weight [23]. BSFFs also contain several micronutrients, Cu, B, Zn, Mn, and Fe [23]. In addition to supplying plant nutrients, insect frass may also provide other benefits, such as improved soil structure and water retention, increased soil microbial diversity, and enhanced plant resistance to abiotic and biotic stresses [24,25,26,27,28].

As another route to enhance overall farm sustainability, in Ireland, cereal farmers are exploring new markets and evaluating alternative and/or historically grown crops [29]. Hemp (*Cannabis sativa* L.; Cannabaceae) has a long history of cultivation in Ireland, and the growing of agricultural hemp for seed or as animal fodder is seeing a resurgence in interest [30,31]. Hemp growers in Ireland are steadily increasing in number and becoming more organized, with the aim to develop an Irish hemp industry aligned with EU CAP, Farm-to-Fork, and wider climate policy objectives (Hemp Federation Ireland; www.hempfederationireland.org accessed on 1 August 2025). Almost all of a hemp plant can be valorised, including the roots, foliage, seeds, and flowers; the products obtained range from fibres, seed oils; and bioactive terpenes, cannabinoids, and biopharmaceuticals. Hemp is a fast growing annual so can also be grown as biomass for the construction industry, energy generation, and carbon sequestration [32,33]. Hemp seeds are rich in fatty acids, including natural omega-3 and omega-6, and hemp protein, derived from the seed cake after seed oil extraction, is used as a food supplement by athletes and fitness enthusiasts [34].

The Irish hemp community is largely driven by organic farmers who have a strong interest in circular economy systems and sustainable farming methods. The overall aims of this project were to form a connection between hemp growing and IFFs by performing a preliminary evaluation of how monoecious and dioecious hemp plants respond to a commercial BSFF produced in Ireland (HexaFrass, Co Meath, Ireland). The specific objectives of the study were to (1) determine how hemp seedlings respond to different application rates of HexaFrass; (2) compare plant responses to HexaFrass with responses to other commercially available organic fertilisers; (3) examine whether monoecious and dioecious cultivars of hemp, and male and female dioecious plants, respond similarly to HexaFrass application; and (4) evaluate the consistency in effects sizes by the repeat testing of HexaFrass under similar growing conditions.

## 2. Materials and Methods

### 2.1. General Methods

Experimental methods were based on protocols developed during previous HexaFrass plant growth studies at University College Dublin [16,17,21]. All trials were carried out in glasshouses located at Rosemount Environmental Research Station (53°18′19.2″ N 6°13′59.5″ W) between March and September 2024. During this period, the mean temperature in the glasshouse was 24 ± 10 °C, and the mean relative humidity was 55 ± 20%. No supplementary lighting was used, and all plants were watered with untreated tap water as required.

The growing media used in all trials was an equal mix of Westland Nutrient Rich Garden Soil, coir fibre (Fruithill Farm, Cork, Ireland), and vermiculite. Previous nutrient analysis carried out on this growing media (Southern Scientific Laboratories, Kerry, Ireland) indicated N-P-K values of 0.3-0.02-0.5 by dry weight [21].

HexaFrass (HF) black soldier fly frass fertiliser was sourced directly from the producer, Hexafly, County Meath, Ireland. HexaFrass is a certified organic fertiliser (Certified Product; Irish Organic Association) that typically contains 60% organic matter, is rich in chitin, and has an N-P-K ratio of approximately 4:2:1. Nutrient analysis has shown that HexaFrass also contains important plant micronutrients such as sulphur (6 g/kg), magnesium (5 g/kg), iron (300 mg/kg), and copper (12 mg/kg). The pH of an aqueous 1:1 HexaFrass solution was found to be approximately neutral at 7.3 [21].

To compare the effects of HF with other standard organic fertilisers, in one trial, we also tested chicken manure (CM) pellets (Westland Organic Chicken Manure Pellets; Woodies Store, Dublin, Ireland; N-P-K 4.5-3.5-2.5) and Miracle-Gro (MG), a complete nutrient organic fertiliser (Miracle-Gro Performance Organics All Purpose Fertilizer; Woodies Store, Dublin, Ireland; N-P-K 8-5-5). These fertilisers were ground in an electric mill and sieved (1 mm mesh) to produce a powdered form similar to that of the powdered HF.

### 2.2. Plant Cultivation and Harvest

Six cultivars of *Cannabis sativa* L. were used in trials: ‘Fedora 17’, ‘Futura 75’, ‘Felina 32’, ‘CFX-2’, ‘CRS-1’ and ‘Estica’. The four cultivars ‘Fedora 17’, ‘Futura 75’, ‘Felina 32’, and ‘Estica’ were sourced from Hemp-It, Beaufort-en-Vallée, France (https://www.hemp-it.coop/ accessed on 1 February 2024). Two cultivars, ‘CFX-2’ and ‘CRS-1’, were certified 1st-generation seeds from Hemp Genetics International, Saskatoon, Saskatchewan, Canada (www.hempgenetics.com accessed on 1 February 2024). The dioecious cultivar CFX-2 was used in all experiments in order to provide both male and female plants and is also considered a multi-purpose cultivar producing good yields of seed, with high-quality oil and good fibre production with respect to animal fodder [35,36].

To produce seedlings for trials, seeds were scattered evenly on the surface of potting mix described above in seed trays (30 cm × 21 cm), covered lightly with growing media, and placed in a heated germinator for three days. The seed trays were then removed from the heated germinator and placed on a standard bench in the climate-controlled greenhouse and acclimatised to the greenhouse environment for three days. For trials, seedlings at the two true leaf stage were transferred to plastic pots (11 cm × 11 cm × 12 cm) containing approximately 1 L of growing media.

In each trial, experimental treatments were arranged on glasshouse benches using a complete random design (https://www.randomizer.org/ accessed on 1 February 2024) and harvested approximately five weeks after being transplanted (6–7 weeks after sowing). Prior to harvest, as an indication of chlorophyll content, SPAD measurements were taken using an SPAD meter (SPAD-502Plus, Konica Minolta, Tokyo, Japan). At harvest, plant height (soil surface to highest plant part) was measured, and shoots were removed by cutting the stem of the plant at the soil surface. Shoot fresh weight (Fwt) was obtained, and then shoots were placed in paper bags and dried in an oven for 72 h at 60 °C. The dried plants were reweighed to obtain shoot dry weight (Dwt) and shoot dry matter (DM; %), calculated as 100 × (Dwt/Fwt).

### 2.3. The Effect of HexaFrass Application Rate on Hemp Shoot Growth

To examine how application rate affected hemp growth, HF was applied at the following rates per pot: 0, 1, 2, 4, 6, 8, 10, 12, 14, 16, 18, and 20 g. Because the high rates made applying the HF as a top-dressing problematic, in this trial the HF was mixed into the potting mix in each individual pot prior to the hemp seedlings being transferred. There were 18 replicate pots of the 0 g controls, and 10 replicate pots for the other application rates. The dioecious cultivar CFX-2 was used in this trial, but it was not possible to distinguish male and female plants when they were transplanted at the two-leaf stage. This resulted in an imbalance in replication across plant sex and HF treatment, which in the extreme case resulted in 1 male plant and 9 female plants in the 14 g HF treatment.

### 2.4. A Comparison of HexaFrass with Other Organic Fertilisers

To compare HF against standard fertilisers, hemp plants were grown using HF, chicken manure (CM), and Miracle-Gro (MG). To standardise application rates in terms of N content, 4 g of HF and CM, and 2 g of MG, were applied as a top dressing to each pot. We applied the fertilisers as a top dressing as this is a recommended application method for all three fertilisers: indeed, both HF and Westland’s CM are produced in a pelleted form, so these products can be spread as a topdressing using standard farm pellet or granule spreaders. A no-fertiliser treatment was also included in this trial to act as a control. The dioecious hemp cultivar CFX-2 was used, and there were between 6 and 9 replicates per fertiliser treatment per plant sex.

### 2.5. The Effect of HexaFrass on Monoecious and Dioecious Hemp Cultivars

To compare the effects of applying HF to different cultivars of hemp, six agricultural hemp varieties were chosen: three monoecious (Fedora 17, Futura 75, Felina 32) and three dioecious (CFX-2, CRS-1, Estica). For each of the monoecious cultivars, there were 20 replicate plants treated with 4 g HF as a top dressing and 20 no-HF control plants. For the dioecious varieties, for each cultivar, there were 12 plants treated with 4 g HF and 12 untreated (0 g HF) control plants. For these dioecious cultivars, as the plants developed, the final replicate numbers in terms of the combinations of HF treatment (0 g vs. 4 g HF) and plant sex (male vs. female) ranged between 2 and 10 plants.

### 2.6. The Effect of HexaFrass on Macro-Nutrient Content of Hemp Shoots

To examine how HexaFrass affected the chemical composition of hemp shoots, hemp seedlings (cv CFX-2) were grown with either 0 g or 4 g of HexaFrass per pot. At harvest, foliage was removed from the plant stem, and then the foliage and stems weighed separately to obtain the Fwt. After drying at 65 °C for 3 d, the stem Dwt and shoot Dwt were obtained for each plant. The dried plant material was ground in an electric mill and passed through a fine sieve. Finally, the shoot samples were analysed by a commercial chemical analysis laboratory (Southern Scientific Laboratories, Kerry, Ireland) to provide measurements of the N, P, K, Mg, and Ca content. Due to imbalances in the number of female and male plants across the two HexaFrass treatments, replicate numbers for tissue chemical analysis ranged from 4 to 9 among the eight distinct groups of samples (2 plant part × 2 plant sexes × 2 HF treatments).

### 2.7. Statistical Analysis

Data were collated and graphs produced using Microsoft Excel (v17, Microsoft, Washington, DC, USA) and all statistical analysis was performed using Genstat software (v24, VSNI Ltd., Hemel Hempstead, UK). In most cases, response variables (Fwt, Dwt, DM, SPAD, etc.) were compared among treatment groups using a general linear modelling approach to account for the imbalance in replicate numbers. Approximate normality of errors and equality of variances were checked visually, and no data transformations were performed. Pairwise comparisons between groups were performed using Fishers Least Significant Differences (*p* < 0.05).

In the trial comparing HF with organic fertilisers, the data for male and female plants were analysed separately. Similarly, in the trial investigating monoecious and dioecious cultivars, the data for males, females, and monoecious plants were analysed separately.

In the examination of HexaFrass application rate on plant performance, relationships between growth response variables (Fwt, Dwt, Ht, Leaf number) were assessed using Pearsons’s correlation coefficient. Polynomial and exponential (asymptotic) curves were fitted to the shoot Fwt, Dwt, and Ht data, and the curves compared using AIC and *r*^2^ values. Polynomial (quadratic) curves were of the form:

*y* = *a* + *b*(HF) + *c*(HF^2^).

where *a*, *b*, and *c* are constants, and HF is the HF application rate in g.

The asymptotic exponential curves were of the form:

*y* = *A* + *B*.*r*^HF^

where *A*, *B*, and *r* are constants, and *A* is the asymptote, *B* is negative, and *r* < 1.

### 2.8. Consistency of Effects Among Repeat Trials and Between Monoecious and Dioecious Cultivars

To examine consistency in effect sizes in repeats of the same experiment, the shoot Dwt data of male and female plants for cultivar CFX-2 treated with 4 g and 0 g were extracted from the four trials described above in Section 2.2, Section 2.3, Section 2.4 and Section 2.5. To examine consistency in effect sizes across monoecious plants and male and female dioecious plants, the shoot Dwt data from the experiment described in Section 2.5 were used.

A standardized effect size parameter, Hedge’s *g*, was calculated following Borenstein et al. [37]:

$$g\;=\;\frac{{\bar{x}}_{HF}-{\bar{x}}_{Control}}{s}J$$

where *s* is the pooled standard deviation calculated as,

$$s=\sqrt{\frac{\left(n_{HF}-1\right)s_{HF}^{2}+(n_{Control}-1)s_{Control}^{2}}{n_{HF}+n_{Control}-2}}$$

*J* is an adjustment for sample size,

$$J=1-\left(\frac{3}{4\left(n_{HF}+n_{Control}-2\right)-1}\right)$$

and $\bar{x}$ = sample mean, *s* = sample standard deviation, and *n* = sample size.

An estimate of the variance of *g* can be estimated as

$$V_{g}=J^{2}\;\left[\frac{n_{HF}+n_{Control}}{n_{HF}\cdot n_{Control}}+\frac{{\left({\bar{x}}_{HF}-{\bar{x}}_{Control}\right)}^{2}}{2\left(n_{HF}+n_{Control}\right)s^{2}}\right]$$

and then the standard error as

$${SE}_{g}=\sqrt{V_{g}}$$

These values of Hedge’s *g* were then tested for heterogeneity by performing a meta-analysis of trial results using a residual maximum likelihood (REML) procedure, which, in addition to a test statistic for heterogeneity (Q), also produced combined estimates of the pooled effect of HexaFrass when assuming the estimates from each trial were fixed or random.

## 3. Results

### 3.1. The Effect of HexaFrass Application Rate on Hemp Shoot Growth

**Figure 1:**
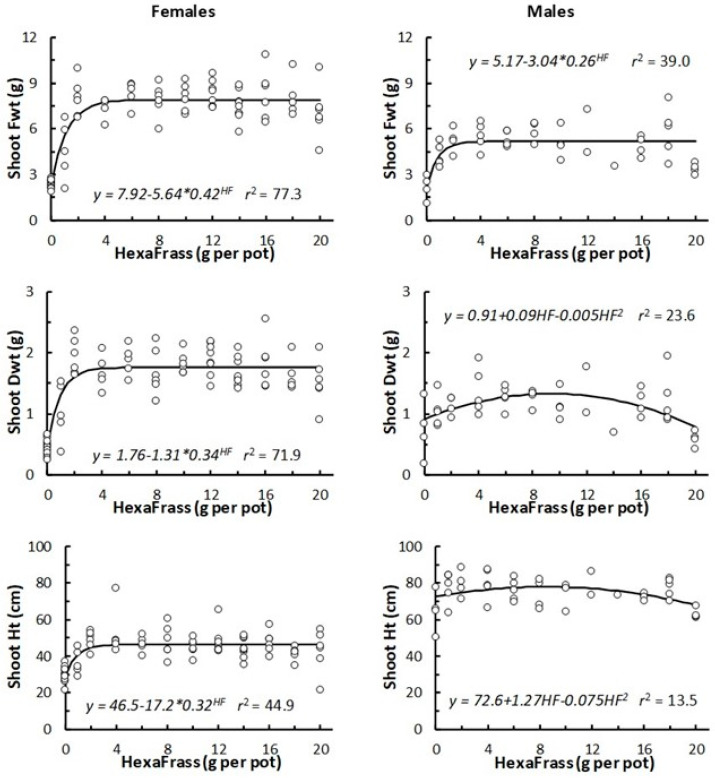
Response of female (n = 81) and male (n = 47) hemp plants (cv CFX-2) to increasing application rates (from 0 to 20 g per pot) of HexaFrass fertiliser. Fitted curves are based on asymptotic exponential models, except for male Ht and Dwt, where polynomial (quadratic) curves were fitted. The *r*^2^ values indicate the proportion (%) of variation in raw data explained by the fitted curve.

The three primary measures of shoot growth (Fwt, Dwt, Ht) all showed strong, positive linear correlations in both female (*r*_p_ > 0.72, n = 81, *p* < 0.001) and male (*r*_p_ > 0.52, n = 47, *p* < 0.001) plants. Consequently, relationships between shoot growth, as measured by Fwt, Dwt, and Ht, and the HF application rate all showed similar trends (Figure 1). For female plants, all three measures of shoot growth exhibited asymptotic relationships with increasing HF rate (Figure 1). Female plants appeared not to show any negative responses in terms of shoot growth or plant mortality even when the highest HF application rates were used (Figure 1). Female shoot size increased steadily with the addition of 1 g and 2 g of HF, but after 4 g, no additional increases in shoot growth were observed with additional HF (Figure 1).

Male plants tended to be taller than female plants, but with lower Fwt and Dwt (Figure 1). In terms of shoot Fwt, the male plants showed a clear increase when 1 g and 2 g HF were applied, similar to the response shown by female plants. However, for shoot Dwt and Ht, the positive effect of HF was not as clear as that shown by female plants, and at the highest rates, shoot Dwt and Ht were similar to those observed in the control (0 g HF) plants. Consequently, the relationships between shoot Dwt and shoot Ht with HF application rate were not clearly asymptotic, and therefore, polynomial (quadratic) curves were more appropriate for these data. In general, the amount of variance explained by the curves fitted to the male data was lower than that for the female plants, as indicated by the reduced *r*^2^ values (Figure 1).

### 3.2. The Response of Hemp to HexaFrass Compared with That Obtained with Other Organic Fertilisers

All of the response measures related to shoot growth (Fwt, Dwt, Ht, leaf number) were increased by HF application in both male and female plants, although the number of leaf pairs and shoot Dwt of male plants were not statistically separated from the no-fertiliser controls (Table 1). For shoot Fwt and Dwt, HexaFrass (HF) produced a growth response similar to that seen with chicken manure (CM) but slightly lower than that achieved with Miracle-Gro (MG) (Table 1). For shoot Ht, HF resulted in a similar response to MG, and, on average, produced slightly taller plants than that seen with CM (Table 1). In terms of shoot quality, Dwt per unit length (DPL) was significantly higher in the fertiliser treated plants than in the controls, with HF producing a similar response to that seen with CM, but slightly lower than that achieved with the MG (Table 1). No obvious patterns were seen for foliage chlorophyll levels (SPAD) and shoot DM (%), and these measurements were not statistically different among the four fertiliser treatments (Table 1).

### 3.3. The Effects of HexaFrass on Shoot Growth in Monoecious and Dioecious Hemp Cultivars

**Figure 2:**
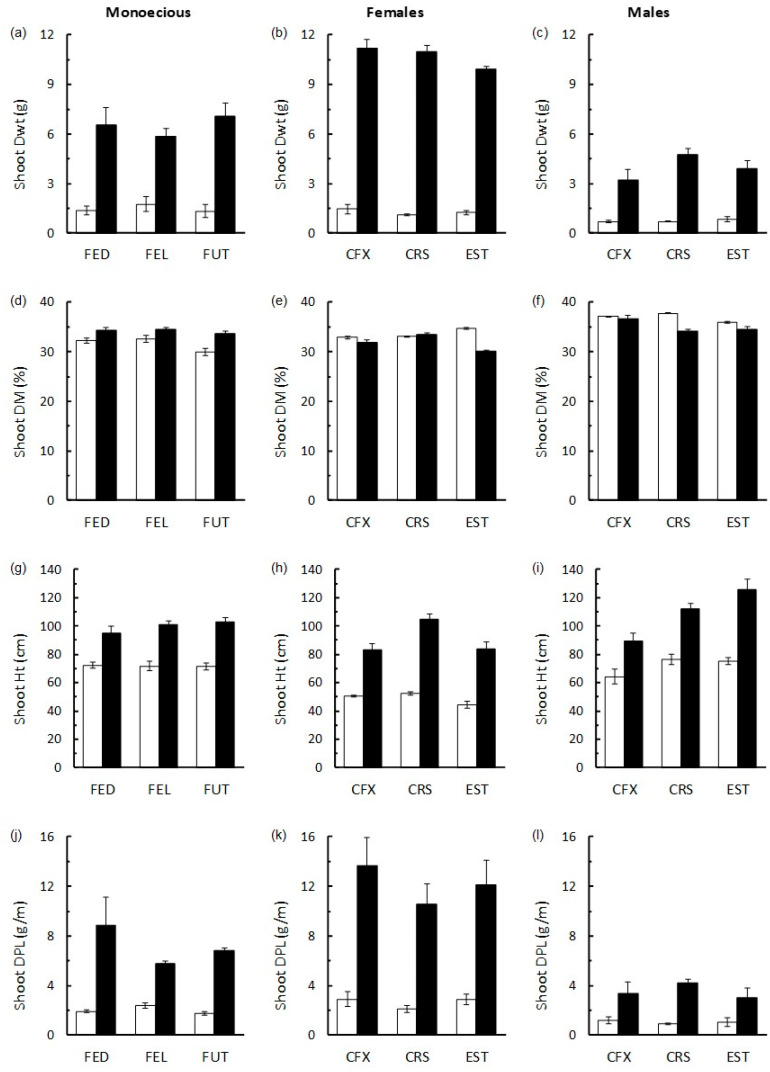
Response of monoecious (**a**,**d**,**g**,**j**), dioecious female (**b**,**e**,**h**,**k**), and dioecious male (**c**,**f**,**i**,**l**) hemp cultivars to addition of HexaFrass (HF) fertiliser (4 g per pot; black bars) compared with no-fertiliser control plants (white bars). (**a**–**c**)—shoot dry weight (Dwt, g); (**d**–**f**)—shoot dry matter (DM; %); (**g**–**i**)—shoot height (Ht, cm); (**j**–**l**)—shoot dry weight per unit length (DPL; g per m). Values given are mean ± SE.

For all three monoecious cultivars, and for both male and female plants in all three dioecious cultivars, the addition of 4 g HF significantly increased Shoot Dwt, Ht, and DPL (ANOVA *p* < 0.001 in all cases; Figure 2). These positive effects were most prominent in dioecious female plants compared with dioecious male and monoecious cultivars. For example, in terms of shoot Dwt, HF caused an 8-fold increase in dioecious females, 5-fold increase in dioecious males, and a 4.5-fold increase for monoecious cultivars (Figure 2).

There was no overall effect of HF on DM (%) for dioecious male (ANOVA, *p* = 0.489) and dioecious female plants (ANOVA, *p* = 0.106). For the monoecious cultivars, HF caused a slight (~8%) but consistent and statistically significant increase in shoot DM content (ANOVA, *p* < 0.001) (Figure 2). The considerable increase in shoot Dwt in the HF treated female dioecious cultivars resulted in these plants having much higher shoot density than the other groups of plants, with an average of over 12 g of dry weight per m of shoot across the three cultivars tested, compared with 7 g per m for monoecious cultivars and 3.6 g per m for the dioecious male plants.

### 3.4. The Effect of HexaFrass on Macro-Nutrient Content of Hemp Shoots

In general, the hemp stems had lower nutrient contents than the hemp foliage, although levels of K in stems and foliage were comparable (Table 2). Plant sex had no significant effect of nutrient content of stems, whereas in leaves the males plants had significantly lower K levels and significantly higher Mg levels compared with female plants (Table 2). Applying 4 g HF significantly increased the P, K, and Mg content of hemp leaves and stems (Table 2). Levels of Ca in both foliage and stems, and in both male and female plants, were not increased by the addition of HF. On average, N content (% dry matter) was slightly lower in plants treated with HF (Table 2).

When considering hemp plants in terms of forage, it is necessary to consider the total nutrient availability, which includes both total dry matter and the percentage nutrient content. So, for example, in the case of N, even though N content was lower in HF treated plants, the stem and leaf dry matter in these treated plants was double that of the control plants (Table 2). Thus, when multiplying the dry matter by the N content (both foliage and stems), the female hemp seedlings treated with HF would supply 25.9 mg N per plant compared with 12.3 mg N in the control plants. Similarly, the male hemp seedlings treated with HF would supply 18.5mg N compared with 10.2mg N in the control plants.

### 3.5. Consistency of HexaFrass Effects on Hemp Shoot Growth

**Figure 3:**
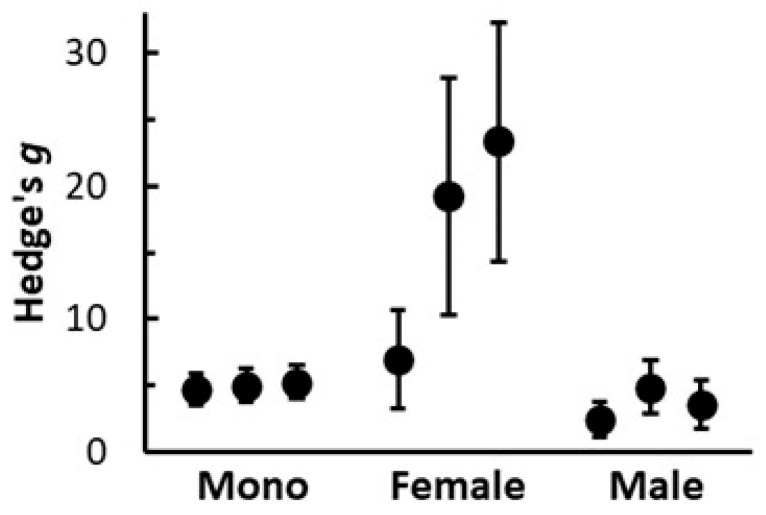
Standardized effect sizes (Hedge’s *g* ± 95% CIs) based on shoot dry weight of hemp when grown with 4 g and 0 g of HexaFrass fertiliser for three monoecious cultivars (Fedora 17, Futura 75, Felina 32) and female and male plants for three dioecious cultivars (CFX-2, CRS-1, Estica).

There was considerable variation in effect sizes when comparing the response of monoecious and dioecious hemp cultivars to HexaFrass. In terms of shoot Dwt, the monoecious hemp plants exhibited very consistent responses to HexaFrass, with Hedge’s *g* ranging from 4.7 to 5.3, and with no significant heterogeneity occurring among the three cultivars (Q = 0.402 for 2 df, *p* = 0.818) (Figure 3). Similarly, the effect sizes for the male dioecious plants had a narrow range of Hedge’s *g* values (from 2.4 to 4.9), again with no significant heterogeneity among the three cultivars (Q = 4.178 for 2 df, *p* = 0.124). The female dioecious plants, however, exhibited a wide range of responses, with Hedge’s *g* values all higher than that obtained for the other groups of plants (from 7 to 23) and showing high heterogeneity among the three cultivars (Q = 14.897 for 2 df, *p* < 0.001). The confidence intervals for the effect sizes for these female dioecious plants within each cultivar were also quite wide, indicating a lack of confidence for these estimates, although this was at least partly due to the low replicate numbers obtained in these groups of plants (two and seven replicates per cultivar per HexaFrass treatment) (Figure 3). Overall, based on shoot Dwt, the female dioecious plants exhibited a considerably stronger response to the application of 4 g HexaFrass compared with male plants and the monoecious cultivars (Figure 3).

**Figure 4:**
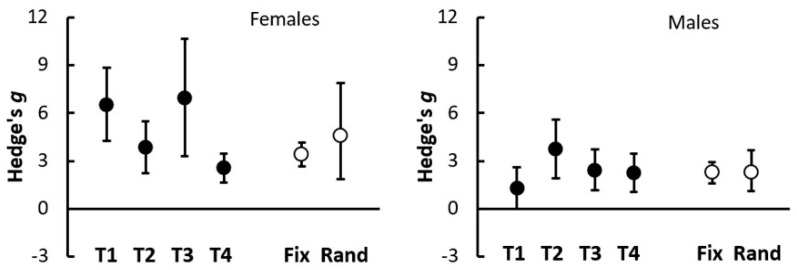
Standardized effect sizes (Hedge’s *g* ± 95% CIs) for four glasshouse trials (T1–T4) comparing shoot dry weight of female and male plants of hemp cultivar CFX-2 when grown with 4 g and 0 g added HexaFrass fertiliser. The fixed and random combined effect size estimates were obtained via a REML meta-analysis procedure using Genstat software.

In terms of within-cultivar consistency, applying 4 g HF increased the shoot Dwt of both male and female CFX-2 hemp plants across four independent trials (Figure 4). In one trial, however (T1; Figure 4), the positive effect was not statistically significant for male plants (Figure 4). In terms of standardized effect sizes based on shoot Dwt, Hedge’s *g* was on average slightly lower and more consistent for male plants (from 1.3 to 3.8) than for female plants (from 2.6 7 to 7.0). Consequently, effect sizes varied significantly among trials for female plants (Q = 14.2 for 3 df, *p* = 0.003) but not for males (Q = 4.6 for 3 df, *p* = 0.201) (Figure 4). Overall combined effect sizes (Hedge’s *g*) were higher for female plants (Fixed 3.43, Random 4.60) than for male plants (Fixed 2.27, Random 2.30) but were all significantly greater than zero (*p* < 0.001), enhancing our confidence in these results (Figure 4).

## 4. Discussion

Overall, our findings indicate that application of the BSF frass fertiliser HexaFrass (HF) significantly increased shoot height and weight of hemp seedlings compared with that achieved in no-fertiliser control treatments. The occurrences of these positive effects were generally consistent, in that they were observed across three monoecious varieties of hemp and in male and female plants of three dioecious varieties. These results align with previous studies where soil amendments created from silkworm (*Bombyx mori* L.) frass also increased the dimensions, growth rate, and yield of hemp shoots [38,39]. Additionally, HF produced similar effects on hemp growth as those obtained with two commercially available organic fertilisers (chicken manure, Miracle-gro). Several previous studies also reported that the application of IFFs produces equivalent effects to standard fertilisers, both in glasshouse trials investigating herbs, vegetables, grasses, and cereals and in field trials using maize [16,17,19,20,21].

The positive effects of HexaFrass on plant performance were observed at relatively low application rates, and the application of additional HF did not result in further hemp shoot growth. Several previous papers have also reported polynomial or asymptotic relationships between hemp growth and fertiliser application rates [40,41,42,43,44]. Yang et al. [45] reported that an increase in applied N generally promoted hemp growth, but excessive supply could reduce nitrogen use efficiency and/ or plants could be weakened by oxidative stress. Similarly, non-linear relationships between plant growth and IFF application rate have been reported for several plants, for example, tomatoes [46], forage plants [17], herbs [16], grasses [19], and cereals [21].

For female plants, shoot Fwt, Dwt, and Ht levelled off at approximately 4 g HF pot, and this application rate was adopted in all subsequent trials. For male plants, there was some indication that at the highest application rates used (18–20 g HexaFrass per pot), plant growth started to be stressed, although we did not observe the pronounced inhibition of shoot growth or plant death as seen in some other studies [21]. Currently, we are unsure of the mechanism behind the lack of additional shoot growth in hemp plants treated with high IFF application rates, but factors such as plant density, root zone compaction, the build-up of bioactive chitin, surplus nutrients, or the production of excess ammonium via mineralisation have all been mooted and require additional research [47,48,49].

It is also possible that applying the total dose of IFF to young hemp seedlings in a single event meant these plants could not utilise all the available nutrients before leaching or breakdown of the fertiliser had occurred. Hemp may respond positively to boosts in nutrients at the flowering, budding, or seed formation stages, and future research should examine the application of IFFs at different life stages and/or compare single versus split IFF application regimes. From the above discussion, if IFFs are to be used in commercial hemp production systems, it would be necessary to ascertain the optimal application rate, and fertiliser regime, in terms of both plant performance and economic considerations.

With a view to using agricultural hemp as livestock forage or as a component of mixed rations, application of HexaFrass increased both the biomass and the nutrient density of foliage and stems. Overall, the hemp seedlings treated with HexaFrass had higher concentrations of phosphorus, potassium, and magnesium in the above-ground tissues, although calcium and nitrogen were not similarly increased. Previously, the application of IFF has been shown to have mixed effects on the nutrient properties of plant foliage. For example, application of IFF has resulted in increased N, P, and K in barley straw [21]; increased N but not P and K in spinach [50]; and increased K in sunflower leaves but with negligible effects on N and P [51]. Although not a primary aim of this investigation, future work would benefit from a more detailed analysis of IFF-treated hemp material in terms of its value as livestock fodder, for example, by quantifying components such as protein, carbohydrates, energy content, and digestible fibre content [52,53].

Although the occurrence of positive effects on shoot growth occurred consistently in all trials, across all cultivars, and for both male and female plants, the *magnitude* of these responses demonstrated both systematic and seemingly random heterogeneity. Previously, differences in the size of growth effects achieved by IFFs have been attributed to factors such as the plant taxa tested, the insect species producing the frass, and the composition of the rearing diet [16,20,24,53]. In our trials, systematic differences in responses to HF were clearly apparent between male and female dioecious hemp plants, in that female plants within each cultivar accumulated greater shoot Dwt than the male counterparts when HF was applied. The male hemp plants were significantly taller than the female plants, and it was generally the case that the female hemp plants were shorter and bulkier than the male plants [54]. The female plants, although shorter, accumulated disproportionate shoot Dwt than the male plants in response to HF application, accentuating the differences in dry weight per unit length of shoot. The phenotyping, and separation of, male and female hemp plants treated or not treated with IFF is of interest, and could be performed using morphological parameters, plant chemistry, or phenological events such as germination, flowering, and seed set.

Even when grown under relatively consistent conditions, using the same cultivars, and using the same growing media, hemp plants can exhibit high levels of phenotypic heterogeneity [55,56]. Previously, Wang et al. [22] reported differences between trial years in the response of rice to BSF fertiliser, and Rodgers et al. [19] described variation among their trials when HF was applied to perennial rye grass. Within a wider framework of reproducibility of scientific results [57], and because of inherent inconsistencies in the quality of organically-derived fertiliser products, repeated, standardized testing is highly desirable to provide information on the generality of effects, the consistency of effect sizes, and, therefore, what might be considered a ‘typical’ response [58,59,60]. The use of standardized effect sizes, such as Cohens *d* and Hedges *g*, can be useful tools in this regard, as they allow between-trial heterogeneity to be quantified within the same study (as in our case), in addition to identification of factors contributing to heterogeneity in effect sizes across different studies [37,61].

We concede that our investigation represents a preliminary study, and that the results presented were obtained from plants grown over a short period, in small containers, under benevolent glasshouse conditions. Nevertheless, several authors have indicated that, as commercial IFFs are relatively novel plant-promoting products, it is necessary to gain initial empirical data across a wide range of plant groups so as to identify commonalities and situations where variation occurs [50,62]. The scaling up of experiments to larger, longer-term trials, utilising a range of soil types and environmental conditions is clearly required to provide additional evidence of the value of IFFs for sustainable hemp production. Additionally, several authors have reported benefits of IFFs centred around enhanced stress resistance and/or activation of defences against invertebrate pests or pathogens [9,26,28,63]. As many of the high-value secondary metabolites produced by hemp are associated with plant defences, the effects of IFFs on terpene and cannabinoid profiles would have high biological and economic relevance. Finally, recent papers have investigated the value of hemp waste as a potential feedstock for the production of black soldier flies and mealworms [64,65]. If IFFs can be used to increase hemp biomass on a commercial scale, and the waste from this hemp is then repurposed as a rearing medium to produce more insect protein and frass-based fertilisers, this would epitomise a circular bioeconomy system with respect to sustainable agricultural production.

## Figures and Tables

**Table 1:** Response of female (Fem) and male (Mal) hemp seedlings (cv CFX-2) to four different fertiliser treatment. Values given are means; sample size ranged between 4 and 9 per group. Residual error indicated by LSD. Cont—controls; HF—HexaFrass; MG—Miracle-Gro; CM—chicken manure. DPL—dry weigh per unit length (g/m). F and *p* values obtained from one-way ANOVA. Within each row, means not sharing the same letter code are significantly different (Fisher’s LSD, *p* < 0.05).

Variable	Sex	CONT	HF	MG	CM	LSD	F	*p*
Fresh Wt (g)	Fem	1.62 ^a^	7.34 ^c^	10.13 ^d^	5.22 ^b^	2.70	21.71	<0.001
	Mal	0.66 ^a^	3.41 ^b^	7.25 ^c^	3.52 ^b^	2.94	9.40	<0.001
Dry Wt (g)	Fem	0.51 ^a^	2.45 ^c^	3.25 ^d^	1.67 ^b^	0.87	22.56	<0.001
	Mal	0.22 ^a^	1.02 ^ab^	2.31 ^c^	1.06 ^b^	0.96	8.92	<0.001
Height (cm)	Fem	20.8 ^a^	53.3 ^c^	52.3 ^bc^	40.8 ^b^	13.8	17.75	<0.001
	Mal	38.2 ^a^	68.1 ^bc^	76.7 ^c^	55.7 ^b^	18.0	9.59	<0.001
Leaf Pairs	Fem	4.6 ^a^	8.1^b^	8.8 ^b^	7.3 ^b^	1.99	11.74	<0.001
	Mal	4.0 ^a^	5.8 ^ab^	8.0 ^c^	6.7 ^bc^	2.23	6.46	0.003
Dry Matter (%)	Fem	31.8	33.4	32.0	30.1	5.63	0.87	0.470
	Mal	32.7	29.7	32.3	27.9	5.58	1.94	0.154
DPL (g/m)	Fem	1.94 ^a^	4.65 ^b^	6.22 ^c^	3.64 ^b^	1.75	12.62	<0.001
	Mal	0.52 ^a^	1.49 ^b^	2.92 ^c^	1.65 ^b^	0.99	10.96	<0.001
SPAD	Fem	36.3	31.7	39.8	36.5	7.82	2.50	0.086
	Mal	24.1	25.6	27.3	30.9	9.82	1.08	0.386

**Table 2:** Chemical composition (% Dwt) and Dwt (g) of foliage and stems of female and male hemp seedlings (cv CFX-2) in response to application of 4 g HexaFrass fertiliser. Values given are means, with residual error indicated by LSD. Replicate numbers for tissue chemical analysis ranged between 4 and 9 for the eight distinct groups of samples (plant part × plant sex × HF treatment). *p* values obtained from unbalanced ANOVA. Within each row, means not sharing the same letter code are significantly different (Fisher’s LSD, *p* < 0.05).

Tissue	Nutrient	Females		Males		LSD	*p*-Value		
		0 g HF	4 g HF	0 g HF	4 g HF		HF	Sex	Int
Leaf	N (%)	1.49	1.50	1.76	1.46	0.59	0.539	0.586	0.367
	P (%)	0.28 ^a^	0.94 ^b^	0.29 ^a^	1.10 ^c^	0.17	<0.001	0.063	0.135
	Ca (%)	5.64	4.99	6.64	6.59	2.49	0.330	0.078	0.679
	K (%)	3.33 ^b^	4.23 ^c^	2.37 ^a^	3.60 ^b^	0.47	<0.001	<0.001	0.238
	Mg (%)	1.53 ^a^	1.88 ^b^	1.82 ^b^	2.10 ^b^	0.31	0.002	0.012	0.742
	Dwt (g)	0.69 ^a^	1.46 ^c^	0.48 ^a^	1.07 ^b^	0.28	<0.001	0.002	0.325
Stem	N (%)	0.65 ^b^	0.44 ^a b^	0.53 ^a b^	0.35 ^a^	0.25	0.027	0.155	0.838
	P (%)	0.14 ^a^	0.67 ^b^	0.11 ^a^	0.54 ^b^	0.24	<0.001	0.180	0.464
	Ca (%)	0.84	0.84	0.94	0.86	0.33	0.649	0.568	0.675
	K (%)	3.13	4.17	3.03	3.75	0.65	0.047	0.443	0.700
	Mg (%)	0.33 ^a^	0.31 ^a^	0.47 ^b^	0.32 ^a^	0.11	0.033	0.093	0.070
	Dwt (g)	0.31 ^a^	0.91 ^b^	0.34 ^a^	0.81 ^b^	0.23	<0.001	0.730	0.377

## Data Availability

Data are available from the corresponding author on request.
